# Supplementary material for: Motility, Biofilm Formation and Antimicrobial Efflux of Sessile and Planktonic Cells of Achromobacter xylosoxidans
Source: Pathogens. 2019 Jan 27;8(1):14. doi: 10.3390/pathogens8010014 (PMC6471707; doi:10.3390/pathogens8010014)
Supplement: Supplementary file 1 [file pathogens-08-00014-s001.pdf]

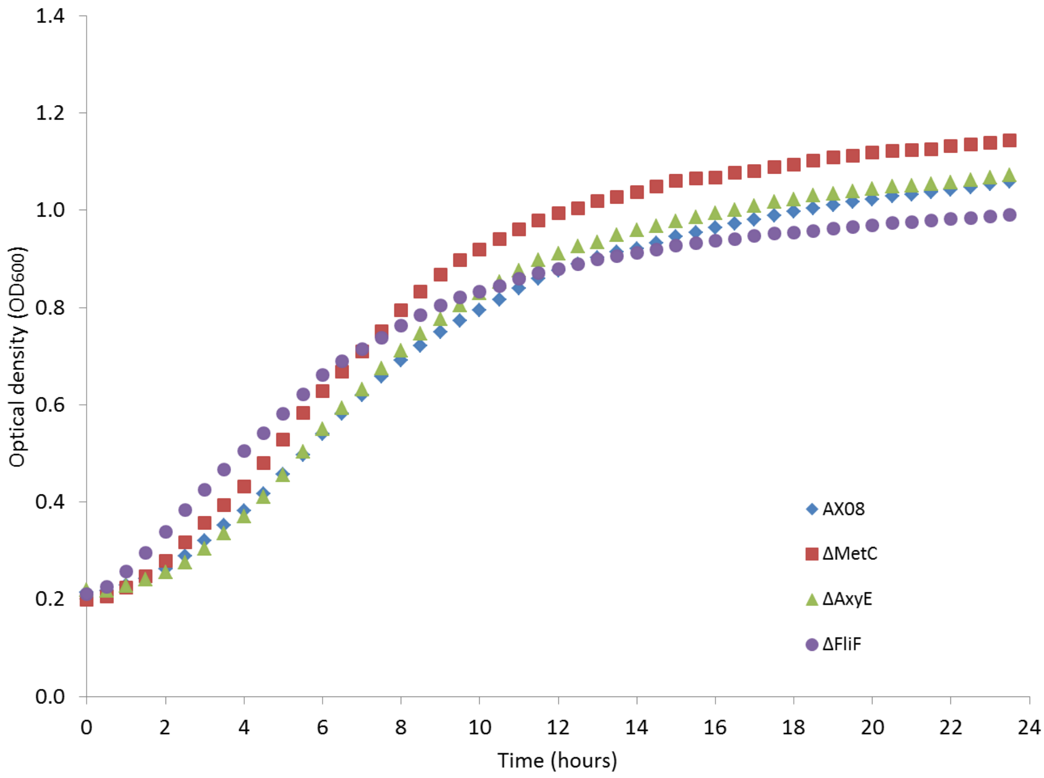

Supplementary figure S1. Growth curves of AX08 (wildtype) and knockout mutants Δ*metC*, Δ*axyE* and Δ*fliF*

Growth rates were determined by optical density measurements at 600 nm in 96 well plates with 200 µL per well. Isolates were cultured in BHI media at 37°C with continuous shaking. Measurements were taken every 30 min using a Multiskan™ GO Microplate Spectrophotometer (Thermo Fisher Scientific San Diego, CA, USA) in kinetic mode.
